# Anomalous Spike Mutations and Sporadic Global Detection of the SARS-CoV-2 BA.2.86 Lineage

**DOI:** 10.31662/jmaj.2025-0118

**Published:** 2025-06-20

**Authors:** Hideki Kakeya

**Affiliations:** 1 Institute of Systems and Information Engineering, University of Tsukuba, Tsukuba, Japan

**Keywords:** SARS-CoV-2, Omicron variant, immune escape, epidemiology, mutation spectrum

## Introduction

During the COVID-19 pandemic, many mutations were observed. Among them, two major genetic leaps stand out: the emergence of Omicron BA.1 and BA.2.86. What was striking was the abrupt appearance of approximately 30 mutations in the spike protein alone, compared to previous variants (Table 1). Though SARS-CoV-2 is an RNA virus, which generally mutates quite often, its mutation rate is relatively slow compared with other RNA viruses ^(1)^. Indeed, the mutation rate of SARS-CoV-2 is 23.9 times slower than that of the Influenza A virus ^(2)^, which makes the emergence of Omicron BA.1 and BA.2.86 all the more conspicuous.

**Table 1.**
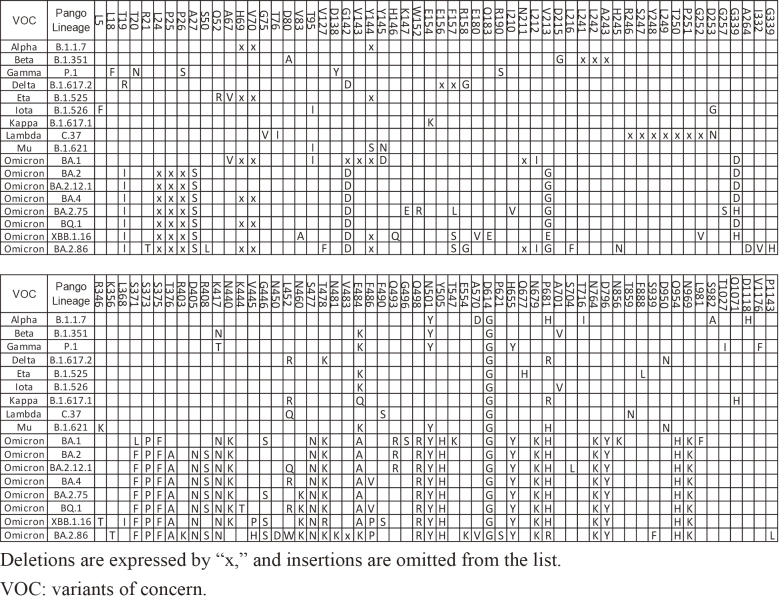
List of Mutations in the Spike Protein for Major SARS-CoV-2 VOCs.

Several hypotheses have been proposed to explain the emergence of the Omicron BA.1 variant ^(3), (4)^. One hypothesis suggests that it slowly evolved through selective pressure in a vaccinated population. Two other primary theories propose either long-term evolution within an immunocompromised individual or mutation in a non-human host before spilling back into humans. However, studies have shown that mutations in immunocompromised patients typically number around 10 or fewer ^(5), (6), (7)^, which is far fewer than the mutations observed in Omicron. As for the theory of evolution in a non-human host, some researchers, such as Wei et al. ^(8)^ and Zhang et al. ^(9)^, have suggested that Omicron could have evolved in mice based on its mutation spectrum and the structure of the receptor-binding domain. However, the original SARS-CoV-2 strain does not infect mice ^(10)^.

While there has been extensive research on the emergence of the Omicron BA.1 variant ^(11), (12), (13), (14)^, fewer studies have focused on the origins of BA.2.86, which represents a mutation shift as significant as the emergence of BA.1. In this study, the author investigates how the BA.2.86 lineage, which is as distant from the preceding variants as the BA.1 lineage was, emerged from both epidemiological and molecular perspectives.

## Materials and Methods

The locations and collection dates of BA.2.86 and BA.2.86.1 registered in the Global Initiative on Sharing All Influenza Data (GISAID) were downloaded in August 2024. The locations of early sample collections of BA.2.86 and BA.2.86.1 were mapped globally to observe chronological and geographical emergence patterns.

For comparison, data on 12 variants (B.1.351, B.1.525, B.1.526, B.1.617.1, C.37, B.1.621, B.2.75, EG.5.1, XBB.1.16, XBB.1.16.16, JG.3, and XBB.2.3) registered in GISAID were downloaded in January 2024. Among these 12 variants, the first six are major variants of concern (Beta, Eta, Iota, Kappa, Lambda, and Mu) with a moderate number of entries. The remaining six variants were selected from those with similar numbers of entries (between 4,500 and 46,000) that appeared between 2022 and 2023. The collection locations of the first 50 samples of BA.2.86.1 and the other 12 variants were compared.

The nucleotide sequences of the Omicron variants BA.1, BA.2, and BA.2.86.1 were downloaded from GenBank in August 2024. Wuhan-Hu-1 (NC_045512.2) was used as the consensus sequence of the original Wuhan strain. The consensus sequences of BA.1 and BA.2 were calculated based on the first 10,000 sequences registered in the National Center for Biotechnology Information (NCBI) GenBank, identifying the most frequent sequence. The consensus sequence of BA.2.86.1 was calculated based on the first 200 sequences without missing reads registered in the NCBI GenBank.

Mutation spectra of the whole genome and the spike genome from BA.2, the closest ancestor of BA.2.86.1, to BA.2.86.1 were calculated based on the consensus sequences. Mutation spectra from Wuhan-Hu-1 to BA.1 were also calculated for reference. To determine the mutation spectrum, dynamic programming matching using Levenshtein distance―with +1 for matches, −1 for mismatches, and −2 for deletions and insertions―was applied to find the best matching alignment.

## Results

**Figure 1.**
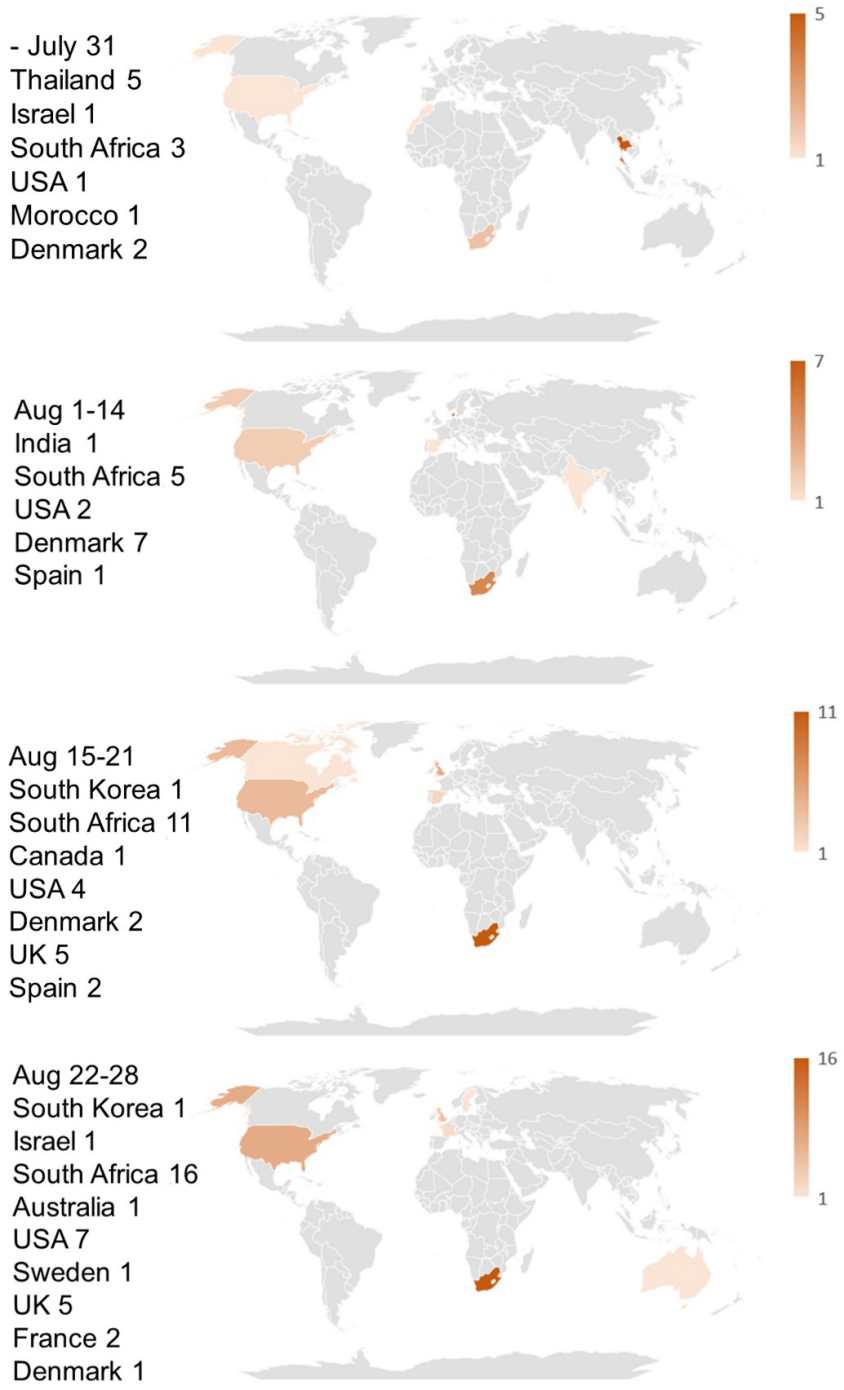
Heatmaps showing the areas where BA.2.86 and BA.2.86.1 were sampled in July and August 2023.

The locations of early sample collections of BA.2.86 and BA.2.86.1 from July 2023 to August 2023 are mapped in Figure 1. As this figure shows, BA.2.86 and BA.2.86.1 infections were observed worldwide from the onset, and no epicenters for these variants are detectable.

The locations of the first 50 collected samples of 13 variants (B.1.351, B.1.525, B.1.526, B.1.617.1, C.37, B.1.621, B.2.75, EG.5.1, XBB.1.16, XBB.1.16.16, JG.3, BA.2.86.1, and XBB.2.3) are listed in Table 2. As shown in this table, most variants have clear epicenters, with collection sites concentrated in specific areas. The divergence in the collection locations of BA.2.86.1 is statistically significant in terms of the number of areas where the first 50 samples were collected. The probability of detecting samples in seven or more areas, as seen in BA.2.86.1, is 0.019 under a normal distribution based on data from the 13 lineages in Table 2.

**Table 2.**
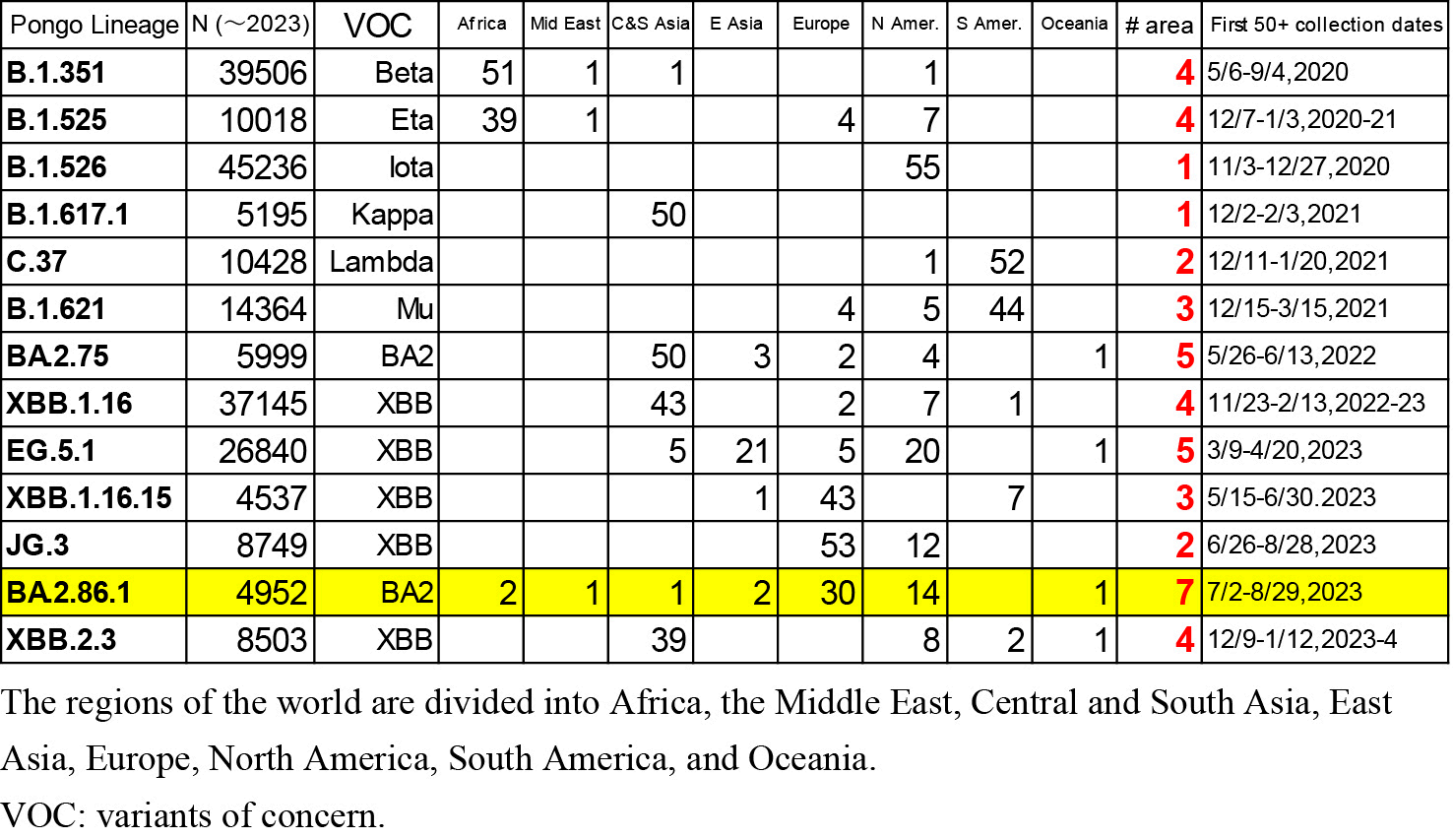
Locations of the First 50+ Sample Collections (the Number Exceeds 50 when Multiple Samples Are Collected on the Same Day).

**Figure 2.**
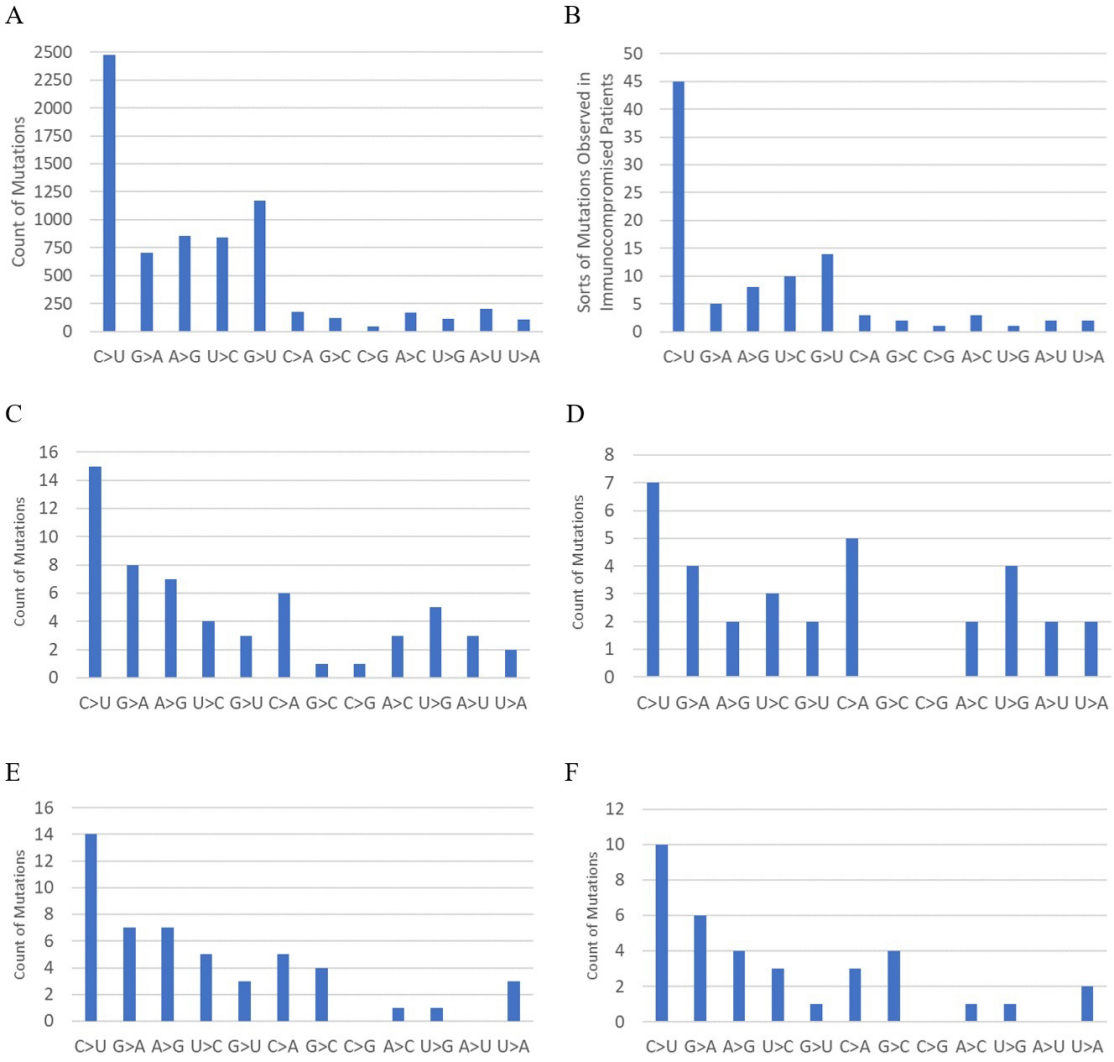
Mutation spectra of SARS-CoV-2 and its variants. A: Mutation spectrum of human SARS-CoV-2 ^(15)^ B: Mutation spectrum of human immunocompromised patients ^(7)^ C: Mutation spectrum of the whole genome from the Wuhan strain to Omicron BA.1 D: Spike mutation spectrum from the Wuhan strain to Omicron BA.1 E: Mutation spectrum of the whole genome from BA.2 to BA.2.86.1 F: Spike mutation spectrum from BA.2 to BA.2.86.1.

Mutation spectra of the whole genome and the spike genome from the original Wuhan strain to BA.1 and from BA.2 to BA.2.86.1 are shown in Figure 2. The mutation spectrum of human SARS-CoV-2 ^(15)^ and that of human immunocompromised patients (types of mutations in reference 7 are used instead of counts of mutations) are also shown for reference. Previous studies have shown that the mutation spectrum from the original Wuhan strain to BA.1 differs significantly from that of human SARS-CoV-2 and is more similar to that of mice ^(8)^. The mutation spectra of the whole genome and spike genome from BA.2 to BA.2.86.1 also differ significantly from that of human SARS-CoV-2, with p values of 0.0073 (whole genome) and 0.010 (spike), based on the G-test, which is used to assess the difference between observed and expected frequencies in categorical data. For reference, the G-test results between distributions C and E, and between D and F in Figure 2 are p = 0.42, and p = 0.29, respectively.

## Discussion

As Figure 1 shows, BA.2.86 and BA.2.86.1, which are quite distant variants from the previous ones, suddenly emerged simultaneously across the globe. Since it is unlikely that similar viruses with the same 30 spike mutations appeared independently from multiple sources, a mechanism must have existed to rapidly spread the same new strain worldwide. BA.2.86 and BA.2.86.1 are not infectious enough to spread as quickly via human-to-human transmission as Omicron BA.1 did at the onset of its emergence, as indicated by the small total number of entries in GISAID. An additional L455S mutation was needed for the new strain (JN.1) to infect a large population globally. Therefore, natural community infection cannot explain the rapid spread of BA.2.86 and BA.2.86.1 across the world.

**Figure 3.**
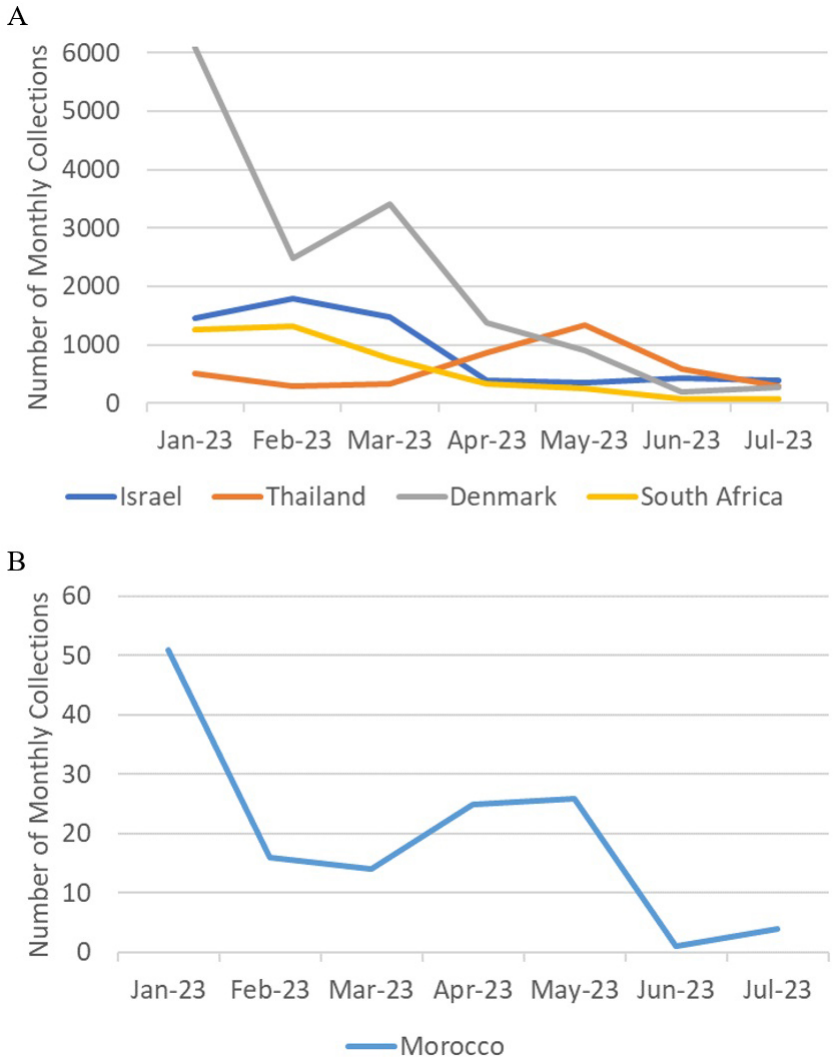
Monthly number of SARS-CoV-2 samples collected in countries where the BA.2.86 lineage was detected in July 2023 (excluding the United States).

Sampling bias cannot account for the sporadic collections of BA.2.86 and BA.2.86.1 either, because among the six countries where the samples were detected in July 2023, the USA is the only country where SARS-CoV-2 was heavily sampled during the same period (17,558 out of 55,963 registrations worldwide), while the number of registered sequences in Israel, Thailand, Denmark, South Africa, and Morocco were 387, 300, 270, 71, and 4, respectively. Since sample collection had been more vigorous in these countries prior to the detection of the BA.2.86 lineage, as shown in Figure 3, its sudden emergence cannot be attributed to limited surveillance in the preceding months. BA.2.86 samples likely would have been detected through these intensive sampling activities if they had been present before July 2023. It is also noteworthy that samples of the JG.3 lineage, which appeared at the same time as BA.2.86, were found in only two areas.

Based on the mutation spectrum of BA.2.86 compared to BA.2, it is most likely that the evolution of BA.2.86 occurred in an animal host other than humans. Immune escape in immunocompromised human patients or in a heavily vaccinated human population can hardly explain the deviation in the mutation spectrum from that of humans. Indeed, the mutation spectrum pattern in immunocompromised patients (Figure 2B) matches that of humans and differs from those of the Omicron variants. Although vaccination rates were high in some of the countries where BA.2.86 first emerged, they were low in others, such as South Africa and Morocco, which are located far from highly vaccinated regions. The possibility of immune escape is further negated by the fact that the neutralization escape of BA.2.86 is similar to that of XBB variants just prior to the emergence of BA.2.86 ^(16), (17)^. Although BA.2, which has a lower ability to escape neutralization, is the phylogenetic ancestor of BA.2.86, immune escape from BA.2 accompanied by sudden massive mutations through unnoticed community spread is unlikely, given the intense surveillance activities prior to the detection of the BA.2.86 lineage, as shown in Figure 3.

Evolution in independent non-human hosts worldwide is also unlikely, as it is improbable that the same 30 mutations occurred simultaneously in multiple locations by coincidence. One possible theory to explain the emergence of a completely new variant, deviating from human evolution, is a lab-grown virus in an animal host. If a viral strain was grown in animal cell cultures, or if lab animals were infected with a new strain of the virus, either deliberately or accidentally, and then sent to collaborating laboratories worldwide for experimental purposes with improper containment, it could lead to the global emergence of the same new virus strain that is completely different from previous variants, with a non-human mutation spectrum. Further investigation is needed to conclusively determine the origin of BA.2.86.

## Article Information

### Conflicts of Interest

None

### Author Contributions

The entire work was performed by Hideki Kakeya, the sole author.

### Approval by Institutional Review Board (IRB)

This research is solely based on the publicly available data and does not involve any human subjects or laboratory animals, requiring no ethical approval to carry out the study.

### Data Availability

The source code used for this study is available at https://visual-media-lab.github.io/data/Mutation_Spectrum/index.html
